# Reliability of Using Motion Sensors to Measure Children’s Physical Activity Levels in Exergaming

**DOI:** 10.3390/jcm7050100

**Published:** 2018-05-02

**Authors:** Nan Zeng, Xingyuan Gao, Yuanlong Liu, Jung Eun Lee, Zan Gao

**Affiliations:** 1 School of Kinesiology, University of Minnesota Twin Cities, Minneapolis, MN 55455, USA; zengx185@umn.edu; 2 Department of Education, East China Normal University, Shanghai 200062, China; xygao@dedu.ecnu.edu.cn; 3 Department of Health, Physical Education and Recreation, Western Michigan University, Kalamazoo, MI 49008, USA; yuanlong.liu@wmich.edu; 4 Department of Applied Human Sciences, University of Minnesota Duluth, Duluth, MN 55812, USA; junelee@d.umn.edu

**Keywords:** pedometers, accelerometers, measurement, physical activity levels, active video games

## Abstract

Objectives: This study examined the reliability of two objective measurement tools in assessing children’s physical activity (PA) levels in an exergaming setting. Methods: A total of 377 children (190 girls, *M_age_* = 8.39, *SD* = 1.55) attended the 30-min exergaming class every other day for 18 weeks. Children’s PA levels were concurrently measured by NL-1000 pedometer and ActiGraph GT3X accelerometer, while children’s steps per min and time engaged in sedentary, light, and moderate-to-vigorous PA were estimated, respectively. Results: The results of intraclass correlation coefficient (ICC) indicated a low degree of reliability (single measures ICC = 0.03) in accelerometers. ANOVA did detect a possible learning effect for 27 classes (*p* < 0.01), and the single measures ICC was 0.20 for pedometers. Moreover, there was no significant positive relationship between steps per min and time spent in moderate-to-vigorous physical activity (MVPA). Finally, only 1.3% variance was explained by pedometer as a predictor using Hierarchical Linear Modeling to further explore the relationship between pedometer and accelerometer data. Conclusions: The NL-1000 pedometers and ActiGraph GT3X accelerometers have low reliability in assessing elementary school children’s PA levels during exergaming. More research is warranted in determining the reliable and accurate measurement information regarding the use of modern devices in exergaming setting.

## 1. Introduction

Prevalence of insufficient physical activity (PA) among children and adolescents serves as a major contributor to non-communicable diseases such as obesity, depression, Type II diabetes, and cardiovascular diseases [1]. As such, a call has been made to gather effective methods of promoting more PA among children in the hopes of maintaining PA participation into adolescence and adulthood. One such method to promote PA levels among children is exergaming (also known as active video games). Exergaming is an emerging technology that requires the players to be physically active while providing performers with the opportunity for health enhancement, thereby thwarting the perception of videogame playing is always sedentary [2]. Compared with traditional PA, exergaming is often perceived as motivating and fun among children because it makes an attractive option in the quest to promote an active and healthy lifestyle within the school settings [3]. Previous studies have found exergaming is a feasible means for children to engage in higher amounts of PA [4,5], and thus exergaming has been integrated into school-based programs to promote children’s PA [6,7,8,9,10]. In addition, exergaming has been utilized for clinical purposes such as pediatric weight management, and improvement on executive functions of children with autism spectrum disorder and with attention deficit hyperactivity disorder (ADHD) [11,12,13,14]. Evidence has shown that exergaming has the potential to serve as an important addition to therapies for children with such conditions. As exergaming is becoming more popular in pediatric clinical setting, valid methods of assessing PA levels in children are critical to understanding the relationship between PA intervention and health promotion.

Nowadays, numerous techniques exist for measuring PA. These techniques can be grouped as observation, self-reported instruments, and motion sensors. Although direct observation is considered a gold standard in measuring PA [15], this may not be feasible in many situations because of expectancy bias, observation effect, and even privacy issues on participants [16]. In addition, self-reported instruments such as questionnaire is recommended not to be used in children below the age of 10 or 11 years, because these children do not have the required cognitive skills which may lead them to over-report their PA levels [17]. Consequently, objective assessment tools such as motion sensors are increasingly being used to assess PA levels in children.

More recently, pedometers and accelerometers have been widely used in assessing children’s PA levels in exergaming settings. For instance, Gao and colleagues [8] used NL-1000 pedometers to monitor children’s step counts and moderate-to-vigorous PA (MVPA) in Dance Dance Revolution (DDR). In addition, a number of studies measured children’s energy expenditure (EE) and time spent in sedentary, light PA, and MVPA utilizing Actigraph GT1M and ActiGraph GT3X accelerometers during exergaming [2,18,19]. These devices are more advantageous because they are not dependent upon individual’s memory, whereas self-reported instruments rely heavily on personal memory to judge the intensity, duration, and frequency of PA. More specifically, pedometers and accelerometers are capable of providing explicit information on the total accumulated quantity of EE and pattern of PA performed throughout the course of the day. As a result, pedometers and accelerometers are perceived more accurate and less subjective as compared to traditional self-reported instruments such as questionnaires and PA logs [20].

In fact, motion sensors have been proven to be reliable in some PA settings. For example, Steeves and colleagues [21] indicated that Omron HJ-303 Pedometer was trustworthy during treadmill running and walking, while the evidence of reliability on GT3X accelerometer was found in assessing activities of daily living [22]. Although many studies have attempted to assess children’s PA levels utilizing pedometers and accelerometers, comparatively little attention is paid to the reliability of using such motion sensors in exergaming settings. To the best of our knowledge, there has been no work on proving reliability of using these motion sensors to measure children’s PA levels during exergaming. Without the evidence of fidelity on these measures, it is difficult to draw conclusions regarding the effectiveness of exergames-based PA interventions. We believe objectively and accurately quantifying the amount of PA in exergaming is important because it will help us better understand the effectiveness of exergames on children’s behavior and health outcomes. Therefore, the purpose of this study was to examine the reliability of using two popular PA monitors (NL-1000 pedometer and ActiGraph GT3X accelerometer) in assessing healthy elementary school children’s PA levels during exergaming.

## 2. Materials and Methods

### 2.1. Participants and Research Setting

A total of 377 (190 girls, 187 boys) first through fourth grade children (aged 6–11 years, *M_age_* = 8.39, *SD* = 1.55) enrolled in a suburban Title I elementary school in West region of the United States. Participants attended two structured PA programs for 150 min every week (30 min × 5 school days). The two PA programs consisted of three 30-min physical education (PE) and two 30-min exergaming classes in the first week (i.e., children went to PE classes on Monday, Wednesday, Friday, and attended exergaming classes on Tuesday and Thursday). In the second week, students played 30-min exergames on Monday, Wednesday, and Friday, and participated in 30-min PE classes on Tuesday and Thursday. The third and fourth week repeated the process of the first two weeks. That is, students were offered equal numbers of PE and exergaming classes every month. This PA pattern was employed throughout the duration of the 18 weeks at school. Children from 20 classes attended the programs with class as the unit. The class size ranged from 17 to 22. The specific inclusion criteria for this study were (1) children were first through fourth grade and aged 6–11 years, (2) without a diagnosed physical or mental disability according to school records, and (3) with parental consent and child assent. Participants in this population were selected because this age range is a critical period for children to develop and maintain a physically active lifestyle.

In this study, children’s PA behaviors only during exergaming were included to determine the reliability of motion sensors in assessing PA. Twelve exergaming stations were set up in a classroom, each station was equipped with eight different Wii exergames including, but not limited to: Wii Fit, Wii Cardio Workout, Just Dance, and Wii Sports. The children were instructed to learn how to play these games prior to the testing (e.g., imitate the movement projected by on-screen avatars). The selected exergames are school-age appropriate that require a variety of body movements such as jumping, kicking, punching, and ducking. A trained teacher supervised the children during exergaming. Each station accommodated up to two children to play, and children rotated from one station to another station and every 8–10 min allowing for a short duration transition. As such, all children in one class had the opportunity to play exergaming simultaneously, and were also able to play different exergames during the program. Prior to initiating data collection, The University Institutional Review Board approved the study protocol and informed consent forms were obtained from guardians and children.

### 2.2. Instruments

The NL-1000 pedometer. The NL-1000 (New Lifestyles Inc., Lee’s Summit, MO, USA) is an advanced waist-worn pedometer that uses a piezoelectric sensor to quantify daily step counts, track the intensity of each step and displaying intensity as MVPA time accumulation [19]. The data of steps and MVPA time can be read directly from the screen, meaning that no downloading or cleaning of data is required. The NL-1000 has been shown to accurately detect steps taken in both laboratory and free-living settings for children [23,24]. In this study, the pedometer data were captured during monitoring period, and steps per min (step counts/30 min) were calculated as the outcome variable.

ActiGraph GT3X accelerometer. The ActiGraph GT3X (ActiGraph Co., Pensacola, FL, USA) is a compact (3.8 × 3.7 × 1.8 cm), lightweight (27 g) and rechargeable accelerometer, which uses a solid-state tri-axial accelerometer to collect motion data on three axes for the highest levels of analytic capabilities available [25]. The ActiGraph GT3X measures and records time-varying accelerations ranging in magnitude from 0.05 to 2.5 G’s. The output is digitized by a 12-bit analog to digital convertor (ADC) at a rate of 30 Hz [25]. The signal passes through a digital filter that band-limits the accelerometer to the frequency range of 0.25–2.5 Hz once digitized [21]. Most recently, researchers have demonstrated acceptable validity and reliability of ActiGraph GT3X when using with children in school and free-living conditions [26,27]. Given a short-duration PA (30 min) of each class and the aims of this study, activity count was set at 1 s epoch length. That is, in-class PA levels were quantified as average activity counts per second (30 Hertz) for the intensities of the activities. PA levels were classified into sedentary, light, moderate, and vigorous categories according to the cut-points set by Evenson and colleagues [28]. The following cut points were applied to define different PA levels (sedentary: 0 to 100; light PA: 101 to 2295; and MVPA: 2296 and above). In the present study, children’s time spent in sedentary, light PA, and MVPA were used as the outcome variables to classify PA intensities.

### 2.3. Procedures

Graduate research assistants were recruited from the University and were trained to assist the program supervisor of the study. Prior to data collection, the research assistants explained the purpose of the study to the participants, including activity sequence and instruction on how to appropriately wear motion sensors. At the start of practice, all children were assigned an identification number that matched up with the number on their pedometers and accelerometers. Each child then was asked to equip a pedometer and an accelerometer that were tied around the waist by an elastic belt and worn on the right side of hip. Although all children in this study had previous experience wearing pedometers and accelerometers in the exergaming program, the research team still assisted the students to make sure all the motion sensors were attached correctly. As such, the reactivity effect was minimized. For each participant, the time was recorded for placement and removal of the pedometers and accelerometers by the program supervisor. The pedometers and accelerometers were collected at the end of the class. Specifically, upon completion of the exergaming session, children took off the belts and reported their step counts pedometer data to the program supervisor and research assistants. In the meantime, accelerometers were retrieved and data were downloaded into ActiLife 6.0 (Actigraph Corps., Pensacola, FL, USA) for data sorting and processing. Data from accelerometers were truncated and matched to the initial time frames when PA occurred for each participant. Finally, all the data were imported into a SPSS data file for descriptive statistical analyses.

### 2.4. Data Reduction and Analyses 

A total of 377 participants took part in the 18-week exergaming program. Due to holidays and other cancellations of class, children actually completed 27 days exergaming session. Among these sessions, day 7, day 11, day 15, day 16, day 22, and day 23 were excluded since they had less than 300 trails. In addition, 188 trails were excluded due to default listwise deletion function of intraclass correlation coefficient (ICC). As a consequence, each participant received 21 repeated assessments. Data were analyzed as follows: First, descriptive statistics for the outcome variables (i.e., standard deviations and means of steps/min, time spent in sedentary, light PA, and MVPA) were performed to describe the data characteristics. Second, the test-retest reliability for outcome variable was estimated using ICC with 95% confidence interval. A two-way mixed effects model was chosen for type of model. Consistency type was selected for the type of index, and single measures were reported. Reliability coefficients were categorized with values less than 0.4 considered poor; 0.41 to 0.6, moderate; 0.61 to 0.8, substantial; and greater than 0.8, excellent reliability [29]. Third, Pearson correlation was computed to estimate the linear relationship between accelerometer (i.e., time engaged in MVPA) and pedometer measurements (i.e., steps per min). Finally, given the present study involved a hierarchically structured data set where the measurements were nested within individual, data were analyzed using Hierarchical Linear Modeling (HLM) [30]. HLM is a flexible approach that can be applied to evaluate inter-individual differences in intra-individual changes over time [31]. That is, HLM separates inter-individual variance from intra-individual so that each participant has his/her own curve. In addition, HLM accounts for the shared variance by multiple observations within the same participant and extends multiple regression to nested or repeated-measures data [32]. Since there were several observations of each person, waves of data were nested within a person. In this study, measurements were nested within students and students were further nested within classes, so three levels were applied to the final analysis. The Level 3 experimental unit was class; the level 2 was student; and the Level 1 was measurement with time spent in MVPA and steps per min as the outcome variables. The ICC and Pearson correlation analyses were performed via the SPSS 20.0 version, and the HLM was conducted using HLM 7.0 software (Scientific Software International Inc., Lincolnwood, IL, USA) for the statistical modeling of thee-level data structures. The alpha level for the present study was set at 0.05.

## 3. Results

### 3.1. Descriptive Analysis

Descriptive results are presented in Table 1. In general, children displayed roughly similar levels of steps per min across all testing days. Additionally, children’s time spent in sedentary, Light PA and MVPA were also quite similar and relatively stable for each repeated measure. Approximately 15 to 20 min valid wearing time were captured by the accelerometers during each 30-min exergaming session. More specifically, the mean steps per min ranged from 23 to 49. Children spent around 9 to 11 min in sedentary, 3 to 6 min in light PA and only accumulated 3 to 4 min of MVPA. In particular, although the last three days had the highest scores of steps per min, students did not show a higher level of time engaged in MVPA.

### 3.2. Intraclass Correlation Coefficient and Pearson Correlation 

The result of ANOVA from ICC demonstrated that there were no differences among trials for accelerometer measurements, *F* (12, 185) = 0.916, *p* = 0.53, indicating that no learning or fatigue effect was detected (Table 2). In addition, ICC for accelerometer assessments showed single measures was 0.033 with a 95% confidence interval from 0.014–0.059, which was considered a low degree of reliability. Furthermore, ANOVA analysis for pedometer assessments did detect a possible learning effect during the 21 classes, *F* (26, 97) = 3.315, *p* < 0.01, but a low degree of reliability was also found between pedometer measurements from ICC, the single measures was 0.199 with a 95% confidence interval from 0.153–0.260. The result of Pearson correlation indicated that there was no significant relationship between time spent in MVPA determined by accelerometers and steps per min determined by pedometers (*r* = 0.027, *p* = 0.597).

### 3.3. Hierarchical Linear Modeling 

In order to further explore the relationship between pedometer and accelerometer assessments by controlling the student background and aforementioned possible learning effect, HLM was employed. We developed the (1) null model, which did not use any predictors to gauge to what extent that a second and third level model could contribute to explain the variance of the outcome variable; (2) control model, which added background variables of students to the null model; and (3) full model, which added the major predictor to the control model. The full model is developed as following:

Level-1 Model:

MVPAmti = *ψ*0ti + *ψ*1ti*(DAYmti) + *ψ*2ti*(TIMEmti) + *ψ*3ti*(SPMmti) + *ε*mti (1)

At level 1 (measurement level), outcome variable was the total MVPA divided by time, control variable “day” represents the “learning effect”, “time” refers to “how long the measurement process took”. The predictor variable, steps per min (SPM), is the pedometer steps divided by time. *ψ*0ti and *ε*mti represented the initial status of student t in class i and a random effect, respectively.

Level-2 Model:

*ψ*0ti = *π*00i + *π*01i*(SEXti) + *π*02i*(AGEti) + *π*03i*(RACE1ti) + e0ti (2)

Level-3 Model:

*π*00i = *β*000 + r00i (3)

Level 3 (classroom level) was added to account for the possible variability among classes, where *β*000 was the grand mean score of all classes, and r00i was the random effect of classes. As a result, steps per min were found to have a significant, positive relationship with MVPA (Table 3). However, only 1.3% more variance at level 1 was explained by adding the steps per min into the full model, which means that the significant association was weak as demonstrated by small proportions of variance explained. Therefore, the results of the HLM implicitly took nesting into account and included more controls in the models, which did not contradict the ICCs results. In other words, the measurement provided by pedometer was not reliable when we assessed students’ MVPA, as a low degree of reliability was also seen in pedometer outputs.

## 4. Discussion

The manufacturer of the motion sensors now offers various instruments for analyzing movement data, but their trustworthiness in exergaming settings has not been documented in the literature. Therefore, this study was designed to provide reliability evidence for two popular motion sensors (NL-1000 pedometer and ActiGraph GT3X accelerometer) that are used as practical and objective PA measurement tools in exergaming settings. Based upon the descriptive analysis, children actually spent most time in sedentary behavior as compared to LPA and MVPA while playing exergames. Although each exergaming session had 30 min, only half to two-third PA time were captured by accelerometers. The results of ICCs showed the estimates of PA levels were inconsistent among children as the ICCs were far from ideal, indicating low reliability of two monitors in measuring elementary school children’ regular PA levels during exergaming play. 

While the findings are unexpected and not accordance with most previous studies indicating these motion-sensors are reliable instruments in assessing children’s PA behaviors in other setting, we identified plausible reason for such discrepancies. In the present study, participants were elementary school children and they were equipped with two motion sensors simultaneously while playing exergames. That is, the inconsistency of instruments placement may lead to data loss (about one-third of PA time missed) and unreliability of motion sensors in capturing movement data in exergaming.

Unlike traditional physical education class where contents are consistently delivered by the instructors, the exergaming activities were carried out by multiple random games. Within a single game console, different games with respective difficult levels can provide varying EE. In our study, students were offered with eight different Nintendo Wii exergames and every student played different types of exergames, thus variability of EE across types of games tended to be large. For example, the “Just Dance” allowed players to perform more than 20 songs consecutively with several difficulty levels depending on how well the player was dancing. Such arrangement resulted in students being exposed to various conditions. Therefore, different types of exergames and game sequence effects might be potential confounding factors on reliability of measurements in exergaming. In addition, children rotated from stations to stations in the present study. That is, when a participant arrived at next station, he or she had to set up a different game to meet his or her interests and needs. In that case, the exergaming was not implemented in a continuous way and children were not really being physically active during these transitions. To this sense, the transition time may lead to inaccurate data because each child would have unequal PA intensities and time of game playing.

The correlation analysis suggested no significant positive relationship between accelerometers and pedometers measures. Furthermore, the HLM analysis further confirmed low reliability of two monitors, which was not contradictory with the ICCs results as very little shared variance could be explained by pedometers and accelerometers. Previous studies using similar activity monitors have shown agreement in measuring PA levels among children and youth [33,34]. These empirical studies utilized different epoch lengths (5 s and 30 s) and cut points to determine sedentary, light PA and MVPA thresholds in either school-based physical education or free-living settings. Although several validated MVPA epoch lengths have been proposed for use in children, the impact of accelerometer epoch length on PA measures remains a contentious issue, particularly with regard to application in school children. For example, researchers suggested that the 15 s epoch length for accelerometers captured more MVPA and sedentary time yet fewer steps and less light PA and total PA were tracked compared to 60 s epoch length [35]. Appukutty and colleagues [36] indicated that the MVPA time were significantly higher with 10 s epoch than 60 s epoch in children. In general, children’s PA patterns tend to be varied and intermittent for differing activities and intensities. The use of shorter epochs would produce higher and more accurate estimates of MVPA time compared to longer epochs [37]. McClain and colleagues [34] further demonstrated that 5 s epoch length in ActiGraph accelerometer yielded the lowest root mean squared error in assessing MVPA, indicating shorter epochs would yield more PA amount. Because of this, children’s PA may be better captured with shorter epochs. In this study, we used 1s epoch length to make sure we accurately captured children’s PA as much as possible. Nevertheless, we still believe that different epoch length thresholds would explain part of the differences between our findings and previous studies. 

Furthermore, it has been noted that different equation should be used when using accelerometers to children with chronic diseases such as congenital heart disease (CHD), cystic fibrosis (CF), dermatomyositis (JDM), juvenile arthritis (JA), inherited muscle disease (IMD), and hemophilia (HE) [38]. For example, Stephen et al. [38] found that agreement between predicted and measured energy expenditure (EE) varied across disease group and ranged from (ICC) 0.13–0.46. With the prediction equation specific to the disease group was presented, an improved range of results (ICC 0.62–0.88) (SE 0.45–0.78) was shown. The researchers concluded that current prediction should be replaced with disease-specific equation in children with chronic conditions as the current equation demonstrate poor agreement. In terms of testing reliability and validity of pedometers in clinical population, it has been reported that pedometers (Yamax SW digi-walker, New Lifestyles NL-2000) are useful for tracking ambulatory movement in the population [39]. For example, in their study with youth with cerebral palsy, NL-1000 pedometer demonstrated an excellent reliability (ICC 0.88–0.99) and validity (ICC 0.78–0.95) with no significant difference between the video step counts and pedometer step counts in walking and running in controlled setting. When using pedometers, however, in population with disabilities, the 3% error cutoff for accuracy may not be realistic for population with disabilities [40].

Finally, it is known that both waist-mounted accelerometers and pedometers have lower accuracy when one’s PA level is at slow speeds [41,42]. For instance, McClain et al. [43] found agreement between NL-1000 and ActiGraph accelerometers estimates of free-living MVPA in 10-year old children, but large differences were also found at other settings, especially when children experienced a low PA intensity. Similarly, although Kinnunen et al. [44] found statistically significant correlations, the overall agreement between pedometer and accelerometer step counts was poor and varied with PA levels. 

The strength of this study lies in that it is the first of its kind to assess the reliability of two motion sensors in measuring children’s PA levels in exergaming settings. However, there are several limitations that should be noted. While multiple sessions of two objective motion sensors were used to explore their reliability, they were not compared to the gold standard measures of energy expenditure. Therefore, a rigorous design and better statistical analyses will be necessary in the future research. In fact, exergaming has several limitations when being considered a PA option. One such limitation is the limited time children spent in being moderately or even vigorously active. Exergaming oftentimes requires the players short bursts of exertion followed by considerable breaks between activities, which may lead to children not very active to the MVPA level. Although there were various difficulty levels available for children in exergames such as beginner, intermediate, and advanced levels, it is implausible to let each child play the advanced level games for every time, because games were selected randomly and varied according to how well the child performed. If children played the same games, they would lose interest in exergaming and the enjoyment of exercise would decrease over time. Therefore, the low speed and random nature of exergaming may result in accelerometers and pedometers having lower reliability in measuring children’s PA levels.

## 5. Conclusions

In summary, the primary finding of the study was that the NL-1000 pedometers and ActiGraph GT3X accelerometers have low reliability in assessing elementary school children’s PA levels during exergaming. More research is warranted in determining the reliable and accurate measurement information regarding the use of modern devices during exergaming. Although the current study does not provide promising reliability evidence for the use of NL-1000 pedometers and ActiGraph GT3X accelerometers in assessing PA in exergaming settings, the findings may render some implications for future exergaming research. First, the same exergaming activities should be employed when motion sensors are used to eliminate the variability caused by different games. Second, researchers should minimize the transition time by increasing the time of game playing to make sure children stay physically active pattern most of the time. Third, placing the monitor on ankles or using heart rate monitors may be more appropriate in assessing children’s PA during exergames play. Finally, researchers should ensure the consistency of the device placement during measurement. When using these motion sensors in clinical population whom might be using exergaming as a therapeutic means, more factors need to be taken into considerations such as type of disease, presence of disabilities, and utilizing disease-specific prediction equation.

## Figures and Tables

**Table 1:** Descriptive statistics for all outcome variables.

Variable	Steps per Min		Sedentary (Min)		Light PA (Min)		MVPA (Min)	
	Mean	SD	Mean	SD	Mean	SD	Mean	SD
Day 1	23.22	15.32	9.69	3.61	3.35	1.72	2.39	2.09
Day 2	36.65	26.25	9.01	3.92	3.73	2.20	3.37	3.21
Day 3	38.40	25.21	10.39	4.3	4.26	2.45	3.31	2.90
Day 4	38.40	27.04	9.61	4.23	4.64	2.59	3.38	2.67
Day 5	42.26	25.24	8.95	3.70	4.70	2.55	3.73	2.94
Day 6	42.24	26.22	9.59	4.45	5.18	2.26	3.95	2.71
Day 7	38.64	23.13	9.61	4.53	5.23	2.36	3.70	2.67
Day 8	40.28	25.74	10.08	4.69	5.44	2.37	3.71	2.53
Day 9	37.57	24.88	9.98	4.56	5.22	2.56	3.69	2.46
Day 10	39.41	27.13	10.77	4.12	5.33	2.66	3.44	2.54
Day 11	40.85	25.28	10.56	4.46	5.46	2.45	3.25	2.49
Day 12	39.19	24.37	10.47	4.37	5.23	2.67	3.32	2.53
Day 13	38.75	23.35	10.73	4.33	5.28	2.60	3.39	2.81
Day 14	37.85	23.01	10.40	3.56	5.40	2.26	3.20	2.16
Day 15	40.29	24.17	10.89	3.95	5.46	2.50	3.19	2.41
Day 16	40.34	25.48	10.93	4.02	5.95	2.66	3.46	2.51
Day 17	36.12	20.82	11.34	4.313	5.41	2.58	3.15	2.14
Day 18	41.60	24.58	9.94	4.17	5.68	2.81	3.48	2.59
Day 19	49.06	29.94	9.64	4.42	6.10	3.04	3.87	2.78
Day 20	47.52	27.80	9.26	3.90	5.60	1.94	3.50	2.13
Day 21	47.50	30.26	9.10	3.96	5.64	2.96	3.69	2.67

SD = standard deviation; Sedentary = time spent in sedentary; Light PA = time spent in light physical activity; MVPA = time spent in moderate to vigorous physical activity.

**Table 2:** Intraclass correlation coefficient (ICC) for accelerometer and pedometer assessments.

		IC	95% CI		*F* Test with True Value 0			
			LB	UB	Value	df1	df2	Sig.
GT3X	Single	0.033 ^a^	0.014	0.059	1.450	185	2220	0.000
	Average	0.310 ^c^	0.155	0.448	1.450	185	2220	0.000
NL-1000	Single	0.199 ^a^	0.153	0.260	7.692	97	2522	0.000
	Average	0.870 ^c^	0.830	0.904	7.692	97	2522	0.000

GT3X = ActiGraph GT3X accelerometer; NL-1000 = NL-1000 pedometer; Single = single measures; Average = average measures; IC = Intraclass correlation; 95% CI = 95% confidence interval; LB = lower bound; UB = upper bound. ^a^ The estimator is the same here, whether the interaction effect is present or not; ^c^ This estimate is computed assuming the interaction effect is absent, because it is not estimable otherwise.

**Table 3:** Hierarchical linear modeling (HLM) for moderate to vigorous physical activity with three-level modeling.

Fixed Effect	Coefficient	Standard Error	*t*-Ratio	Approx. *d.f.*	*p* Value
For INTRCPT1, *ψ*_0_ For INTRCPT2, *π*_00_	-	-	-	-	-
	-	-	-	-	-
INTRCPT3, *β*_000_ For SEX, *π*_01_	0.072128	0.030573	2.359	19	0.029
INTRCPT3, *β*_010_ For AGE, *π*_02_	−0.000908	0.011431	−0.079	343	0.937
INTRCPT3, *β*_020_ For RACE1, *π*_03_	−0.006521	0.003386	−1.926	343	0.055
INTRCPT3, *β*_030_ For DAY slope, *ψ*_1_ For INTRCPT2, *π*_10_	0.032761	0.008744	3.747	343	<0.001
INTRCPT3, *β*_100_ For TIME slope, *ψ*_2_ For INTRCPT2, *π*_20_	−0.002019	0.000476	−4.24	5625	<0.001
INTRCPT3, *β*_200_ For SPM slope, *ψ*_3_ For INTRCPT2, *π*_30_	0.004824	0.001595	3.024	5625	0.003
INTRCPT3, *β*_300_	0.001793	0.000145	12.38	5625	<0.001

SPM = steps per min.
